# The Epithelial-to-Mesenchymal Transition in Cancer

**DOI:** 10.3390/cancers10020052

**Published:** 2018-02-16

**Authors:** Joëlle Roche

**Affiliations:** Université de Poitiers, UMR-CNRS 7267, Laboratoire EBI, SEVE, F-86073 Poitiers, France; joelle.roche@univ-poitiers.fr

The epithelial-to-mesenchymal transition (EMT) occurs during normal embryonic development, tissue regeneration, organ fibrosis, and wound healing. It is a highly dynamic process, by which epithelial cells can convert into a mesenchymal phenotype. However, it is also involved in tumor progression with metastatic expansion, and the generation of tumor cells with stem cell properties that play a major role in resistance to cancer treatment [1,2,3]. EMT is not complete in cancer cells, and tumor cells are in multiple transitional states and express mixed epithelial and mesenchymal genes. Such hybrid cells in partial EMT can move collectively as clusters, and can be more aggressive than cells with a complete EMT phenotype [4]. EMT is also reversible by the mesenchymal-to-epithelial transition (MET), thought to affect circulating cancer cells when they reach a desirable metastatic niche to develop secondary tumors. The EMT process involves the disruption of cell–cell adhesion and cellular polarity, remodeling of the cytoskeleton, and changes in cell–matrix adhesion. It is associated with improvement in migratory and invasive properties. In cancers, EMT inducers are hypoxia, cytokines, and growth factors secreted by the tumor microenvironment, stroma crosstalk, metabolic changes, innate and adaptive immune responses, and treatment with antitumor drugs. Switch in gene expression from epithelial to mesenchymal phenotype is triggered by complex regulatory networks involving transcriptional control with SNAI1 and SNAI2, ZEB1 and ZEB2, Twist, and E12/E47 among transcriptional factors, non-coding RNAs (miRNAs and long non-coding RNAs), chromatin remodeling and epigenetic modifications, alternative splicing, post-translational regulation, protein stability, and subcellular localization [5]. EMT is becoming a target of interest for anticancer therapy [6]. However, more knowledge about the role of EMT in metastasis, its control, and its reversion is necessary. Indeed, alternative modes of dissemination, colonization via a MET-independent pathway, and investigation of circulating cancer cells in the blood support a more nuanced view of the role of EMT and MET in cancer metastasis.

This current Special Issue entitled “Epithelial to Mesenchymal Transition in Cancer” sheds more light on EMT in several selected cancers, and the first six reviews describe the pathology, EMT inducers and their corresponding pathways, EMT involvement in metastasis and drug resistance, and therapeutic perspectives [7,8,9,10,11,12]. The first review by Thierauf et al. describes the most recent findings on the clinical relevance of a mesenchymal-like phenotype for head and neck cancer patients, including more rare cases of mucosal melanoma and adenoid cystic carcinoma [7]. Among classical EMT actors, the abnormal expression in head and neck squamous cell carcinoma (HNSCC) of both the pluripotency-associated transcription factor SOX2 (sex determining region Y-box 2) and of the Kallikrein-related peptidase 6 (KLK6) is discussed. This is followed by a review by Fedele et al. that recapitulates the main endogenous molecular signals involved in the acquisition of the mesenchymal phenotype in metastatic breast cancer, mainly basal-like and claudin-low subtypes that belong to the group of triple-negative breast cancer (TNBC), and their cross-talk with paracrine factors [8]. The importance of the extracellular matrix, paracrine mechanisms, and exosomes is underlined. In the next review, Klymenko et al. describe the EMT and the way in which epithelial ovarian cancer (EOC) metastasizes [9]. Unlike most epithelial malignancies which metastasize hematogenously, EOC metastasis occurs primarily via transcoelomic dissemination, and this exceptional microenvironment is highly permissive for phenotypic plasticity. Current knowledge on EOC heterogeneity in an EMT context is highlighted. Computational modeling of EMT is also presented. This review is followed by a description of EMT in non-small cell lung cancer (NSCLC) by Legras et al.—a cancer that is the major cause of cancer-related death in developed countries [10]. The authors report the role of the transcription factor TWIST1 in EMT in cells with epidermal growth factor receptor (EGFR) mutation and its association with low disease-free survival in EGFR-mutated lung adenocarcinoma patients. A large part of this overview is dedicated to EMT regulation by microRNAs. Next, EMT is described by Gaianigo et al. in pancreatic cancer (PC), which has a poor prognosis due to metastatic dissemination in the very first stages of tumor development mainly attributed to EMT [11]. The focus of this review is the involvement of EMT in treatment resistance. Among EMT actors, the authors give special attention to mutated RAS and the nuclear transcription factor yes-associated protein 1 (YAP1), one of the main transducers of the Hippo pathway. The authors also develop the importance of the large fibrotic tumor microenvironment called desmoplasia, which contributes to PC advancement, chemoresistance, and reduced drug delivery. The role of inflammation, microbiota, and treatment resistance related to EMT are also discussed. The review by Vu et al. summarizes EMT in colorectal cancer (CRC) [12]. In addition to classical transcription factors, the authors describe deeper PROX1 homeobox and forkhead box (FOX) transcription factors, their signaling pathways, several novel EMT inducers in colorectal cancers including neuropilin-2 (NRP2, a receptor for cell guidance molecule and growth factors), and downregulation of several microRNAs. Furthermore, the role of the EMT in circulating tumor cells (CTCs) is also discussed with the possible use of the mesenchymal markers as potential biomarkers for metastasis.

The following review by Grelet et al. focuses on specific aspects of EMT in cancers and cell lines, the molecular mechanisms regulating the TGF-β-induced EMT, and their implications in tumor metastasis [13]. The authors discuss the recent reports emphasizing the regulatory functions of non-coding RNA (microRNAs and lncRNAs, piRNAs, snRNAs, snoRNAs, circRNAs). In the second part of their survey, the authors provide experimental evidence of change in the expression of several tRNAs during TGF-β treatment of the human lung cancer cell line A549.

The third series of reviews addresses more specific mechanisms involved in EMT regulation [14,15,16,17]. First, Roche et al. present epigenetic regulation of EMT mainly in lung cancer with recent data on EZH2 (enhancer of zeste 2 polycomb repressive complex 2 subunit) that behaves as an oncogene in lung cancer associated with gene repression [14]. EZH2 is the catalytic subunit of the PRC2 (polycomb group PcG), which methylates lysine 27 of histone H3, inducing a repressive transcriptional mark. The authors mention that EZH2 inhibitors are under development with clinical trials ongoing. Next, Fu et al. describe the role of the Polo-like kinase 1 (PLK1)—a serine/threonine kinase—in EMT and tumor invasion [15]. Its overexpression correlates with increased cellular proliferation and poor prognosis in cancer patients. To its canonical role in almost every stage of cell division and cytokinesis execution, modulation of DNA replication, and cell survival, overexpressed PLK1 orchestrates EMT and associated events (such as invasion and therapeutic resistance) in tumor cells via the MAPK pathway by directly binding and phosphorylating CRAF (a member of the Raf kinase family), and through AKT or FoxM1-dependent pathways. The therapeutic use of PLK1 inhibitors is discussed. In the following review, Blackwell et al. describe exosomes that are important mediators of intercellular signaling and EMT, with resultant transformation of cancer cells to a more aggressive phenotype [16]. By concentrating proteins or RNA, exosomes may transform nearby cells, as an explanation for the “field effect” phenomenon. In addition, cancer-derived exosomes exert pro-angiogenic effects on epithelial cells. Then, Lu et al. review another interesting aspect of EMT events: the gain of multiple pericyte markers by cells undergoing EMT—called the epithelial-to-pericyte transition (EPT), possibly mediated by the serum response factor (SRF) [17]. These mesenchymal cells phenotypically and functionally resemble pericytes. They are attracted to blood vessels via paracrine PDGF signaling and associate to blood vessels through N-cadherin. EPT cancer cells associate with and stabilize blood vessels to fuel tumor growth, and EPT may promote therapy resistance to antiangiogenic agents. The authors propose a model for EPT regulation.

Next, Forte et al. address concerns about 2D cellular models to study EMT and strongly recommend in vitro tissue-derived cell spheroid models [18]. These three-dimensional (3D) cell culture systems—whose phenotype has been shown to be strongly dependent on TGF-β-regulated EMT/MET processes—present the advantage of exposing cells to more physiological conditions in a microenvironment where cell–cell and cell–matrix interactions are present, and recapitulate in vitro the hypoxic micro-environment of tissue stem cell niches and their formation. The authors explore the mechanistic correspondence in vivo and the possible pharmacological perspectives.

The last review by Jia et al. illustrates the concept of “landscape” previously defined by Waddington in the epigenetic field [19]. Each cell phenotype is considered as an “attractor” which is characterized by a unique gene expression pattern, and determined by interactions between multiple molecular players buffered against environmental fluctuations. Here, the authors illustrate how phenotypic plasticity in cancer cells enables them to acquire hybrid phenotypes during EMT. The perspective of this review is intended to encourage the exchange of ideas between cancer biologists and physicists interested in exploring the physics of biology.

In summary, this special issue covers EMT in several types of cancer, and reviews different actors in EMT regulation with potential therapeutic applications. More work is needed to advance this complex field with the best available models.
