# Erratum: Roche, J. The Epithelial-to-Mesenchymal Transition in Cancer. *Cancers*, 2018, *10*, 52

**DOI:** 10.3390/cancers10030079

**Published:** 2018-03-19

**Authors:** Joëlle Roche

**Affiliations:** Laboratoire EBI, SEVE, UMR-CNRS 7267, Université de Poitiers, F-86073 Poitiers, France; joelle.roche@univ-poitiers.fr

The author wishes to make the following correction to the paper [1]. The following paragraph and the corresponding reference were added [2] (as reference 16 in the *Editorial*):

“Next, Duchemin-Pelletier et al. present data about the role of CK2β, the regulatory subunit of the ubiquitous protein kinase CK2 [16]. In MCF10A mammary epithelial cells where CK2β expression was downregulated, the authors provide evidence that CK2β downregulation can promote the acquisition of characteristics commonly associated with the cancer stem cell phenotype. They demonstrate that a CK2β level establishes a critical cell fate threshold in the control of epithelial cell plasticity. Since EMT supports the induction stem-cell phenotype, identifying the CK2 substrates will improve the discovery of new specific markers for breast cell stemness.”

The reference numbering was also corrected.

The author would like to apologize for any inconvenience caused. The manuscript will be updated and the original will remain online on the article webpage.
